# Accelerated Molecular Dynamics Applied to the Peptaibol Folding Problem

**DOI:** 10.3390/ijms20174268

**Published:** 2019-08-30

**Authors:** Chetna Tyagi, Tamás Marik, Csaba Vágvölgyi, László Kredics, Ferenc Ötvös

**Affiliations:** 1 Department of Microbiology, Faculty of Science and Informatics, University of Szeged, Közép fasor 52, H-6726 Szeged, Hungary; 2 Doctoral School of Biology, Faculty of Science and Informatics, University of Szeged, Közép fasor 52, H-6726 Szeged, Hungary; 3 Institute of Biochemistry, Biological Research Centre, Temesvári krt. 62., H-6726 Szeged, Hungary

**Keywords:** accelerated molecular dynamics, alamethicin, membrane, peptaibol

## Abstract

The use of enhanced sampling molecular dynamics simulations to facilitate the folding of proteins is a relatively new approach which has quickly gained momentum in recent years. Accelerated molecular dynamics (aMD) can elucidate the dynamic path from the unfolded state to the near-native state, “flattened” by introducing a non-negative boost to the potential. Alamethicin F30/3 (Alm F30/3), chosen in this study, belongs to the class of peptaibols that are 7–20 residue long, non-ribosomally synthesized, amphipathic molecules that show interesting membrane perturbing activity. The recent studies undertaken on the Alm molecules and their transmembrane channels have been reviewed. Three consecutive simulations of ~900 ns each were carried out where N-terminal folding could be observed within the first 100 ns, while C-terminal folding could only be achieved almost after 800 ns. It took ~1 μs to attain the near-native conformation with stronger potential boost which may take several μs worth of classical MD to produce the same results. The Alm F30/3 hexamer channel was also simulated in an *E. coli* mimicking membrane under an external electric field that correlates with previous experiments. It can be concluded that aMD simulation techniques are suited to elucidate peptaibol structures and to understand their folding dynamics.

## 1. Introduction

With the growing incidence of antibiotic resistance all over the world, the scientific community is, more than ever, desperate to identify novel, fail-safe ways of treatments for a plethora of devastating diseases. This search is not limited to human pathogens but has also been extended to the problem of agricultural pathogens. The class of antimicrobial peptides (AMPs), defined as short, host-defense peptides found in all life forms, is a promising solution. Many AMPs have already crossed over to clinical trials as novel therapeutics, immunity modulators and wound healing promoters [1]. In this study we focus our attention to a special class of fungal AMPs, known as “peptaibols”, owing to their constituent non-standard amino acid residues like α-aminobutyric acid (Aib) and C-terminal aminoalcohols etc. Peptaibols are produced as secondary metabolites and show microheterogeneity. The diversity in their sequence length, hydrophobicity, antimicrobial properties and producing fungal species contribute to a plethora of peptaibols that are yet to be discovered and studied. The increasing gap between known peptaibol sequences and their three-dimensional structures can be reduced using computational modeling and molecular dynamics simulations. The knowledge of peptide structural dynamics is a key to unraveling the mechanisms of their antimicrobial action. In this study we focus on the use of accelerated molecular dynamics (aMD) simulations to obtain conformational ensemble and structural dynamics of alamethicin F30/3 (Alm F30/3). aMD is an enhanced sampling technique that “boosts” the system over energy barriers, thereby fastening the peptide folding process [2,3]. We also show that aMD can be applied to peptide-membrane systems to study the mode of action without introducing significant errors. Alm F30/3 is a 20-amino acid residue long peptide which belongs to the popularly known class of peptaibols, a group of fungal secondary metabolites. Owing to its antibacterial and antifungal activities, a result of its ion-channel forming property, it has been touted as a model system to study AMPs. The majority of Alm sequences consist of hydrophobic residues, the non-proteinogenic residue α-aminoisobutyric acid (Aib), an acetylated N-terminus and the C-terminal phenylalaninol (Pheol). 

The discovery of Alm is credited to Meyer and Reusser [4], where it was referred as “antibiotic U-22324” obtained from ‘*Trichoderma viride*’ and classified as a cyclic peptide due to its inability to react with ninhydrin. The anti-bacterial activity against gram-positive strains was highlighted. The correct producer was reidentified later to be *T. arundinaceum* from the Brevicompactum clade of genus *Trichoderma* [5]. 

The first possible primary structure of Alm was reported by Payne et al. [6], who described it as a cyclic molecule by linking the γ-carboxylate group of the glutamic acid residue to the first proline in the sequence. The sequence was Pro-Mea-Ala-Mea-Ala-Gln-Mea-Val-Mea-Gly-Leu-Mea-Pro-Val-Mea-Mea-Glu-Gln, where Mea is α-aminoisobutyric acid or Aib. They hypothesized a stack-like tunnel formation by the cyclic Alm structure with hydrophobic interior. Martin and Williams [7] later corrected it by describing the structure of Alm as a linear polypeptide by NMR spectroscopy. The new sequence described by them included an acetylated Aib residue at the N-terminus and a phenylalaninol as a side-chain of the 18th Gln residue. They stressed upon the importance of linear Alm structure to be long enough to stretch across a lipid bilayer and rejected the idea of stacked-ring pores. The presence of proline in the 14th position introduces a slight bend in the structure as shown by X-ray crystallography, NMR and optical spectroscopy [8,9,10]. The helix is formed in such a manner that the polar residues are arranged on one side. Alm is classified as amphipathic due to distinct hydrophobic and hydrophilic faces, which renders the ability to either interact with a membrane horizontally or form voltage-gated ion channels with a vertical insertion. The recent studies to understand Alm conformation and pore formation have been reviewed by Leitgeb et al. [11] and Kredics et al. [12]. Here we provide a short update on various research undertaken on Alm since 2014. The following literature review on Alm is a summary of recent work on the common molecule. Most of these studies were carried out to understand and visualize pore formation in various membranes which formed the basis of our work to study the hexameric Alm pore model embedded in *E. coli* membrane mimic.

Pieta et al. [13] described the pore formation of Alm in a monolayer of 1,2-dimyristoyl-sn-glycero-3-phosphocholine/phosphoglycerol (DMPC/PG) lipids using electrochemical scanning tunneling microscopy (EC-STM). PG lipids constitute the membrane architecture of gram-positive bacteria [14] and the negatively charged PG heads provide the electrostatic surface potential promoting insertion of amphipathic Alm into the membrane [15]. Pieta et al. [13] successfully showed the formation of flower-like hexagonal pattern of six Alm molecules, which supports the barrel-stave model of ion channel formation. Rahman et al. [16] reported the thermodynamics of Alm F30/3 pores ranging from monomer to nonamer of 5 to 11 Å radius in an implicit membrane model. The trimer and tetramer formed 6 Å closed pores whereas the hexamer and octamer formed 8 Å and 11 Å open pores, respectively. Interestingly, all pores formed beyond the pentamer with comparable energy supported the multiple conductance level theory.

Castro et al. [17] also studied the various noncanonical disubstituted amino acids in Alm by replacing one or more Aib residue. Few such substitutions proved to be better alternatives than Aib for inducing well-defined α-helical structures and thermodynamic stabilization. Salnikov et al. [18] characterized a pentameric channel assembly formed by a fluorine-labeled Alm derivative when reconstituted into phospholipid bilayers (POPC) with a peptide/lipid ratio of 1:13. When the ratio was lowered to 1:30, Alm was found to be in dimeric form. The pore formation of Alm has also been studied extensively using computational models. For example, 14 µs long all-atom MD simulations performed by Perrin Jr. et al. [19] revealed that the hexamer pore equilibration was achieved only after 7 µs which remained stable onwards. Another simulation with 10 surface-bound Alm showed membrane binding, but no insertion event was observed for 5 µs. However, simulations at higher temperatures and under an electric field resulted in peptide insertion within 1 µs. Madsen et al. [20] showed that incorporation of silicon-containing amino acids in Alm introduced a 20-fold increase in membrane permeability. Similarly, Das et al. [21] discussed an effective strategy to improve antibacterial activity of peptaibols by capping the N-terminal with 1,2,3-triazole, and with hydrophobic substituents on C-4. Afanasyeva et al. [22] showed that Alm on a POPC membrane, in low concentrations below the threshold, can capture and rearrange free fatty acids which may negatively affect membrane physiological functions. 

Recently, Su et al. [23] studied Alm in a 1,2-di-O-phytanyl-*sn*-glycero-3-phosphocholine (DPhPC) bilayer using electrochemical impedance spectroscopy (EIS) and photon polarization modulation infrared reflection absorption spectroscopy (PM-IRRAS) to understand membrane stability and Alm conformation in the bilayer. They demonstrated that at potentials where the bilayer is stable, the Alm peptides assume a surface-bound state with their helices at a large angle from the bilayer normal. Alm peptides are inserted when negative potentials are applied. The ESI data showed the exact potentials at which the helices are inserted and form an open or closed pore, which indicates that the open/closed ion states are potential regulated. Forbrig et al. [24] also observed the mechanism of Alm ion channel formation in lipid membranes tethered on electrodes through surface-enhanced infrared absorption (SEIRA) spectroscopy. It revealed that the peptide monomers interact with each other before transmembrane insertion. Upon evaluation of Alm orientation based on mathematical models, it was proposed that ion-channels may be formed either by N-terminal integration as monomers or as parallel oligomers. Abbasi et al. [25] visualized the formation of Alm pores in floating phospholipid membrane on gold electrodes, which confirmed the hexamer ion channel formation with the diameter of a pore calculated to be 2.3 ± 0.3 nm. A distribution of various Alm aggregates also confirmed the multiple conductivity states theory. Syryamina et al. [26] described Alm (Alm F50/5 spin-labeled analogs) channel formation in POPC membranes using pulsed electron-electron double resonance (PELDOR) spectroscopy, which revealed that it assembled into dimers and higher oligomers can be obtained by increasing peptide concentration. They hypothesized that dimer formation and peptide reorientation must take place simultaneously and is probably the first step in peptide assembly.

Zhang et al. [27] proposed encapsulation of Alm within a γ-cyclodextrin in a way that its hydrophobic residues are buried in the hydrophobic cavity of γ-cyclodextrin, thereby, improving its solubility in aqueous medium, thermal and pH stability and significantly increasing its antimicrobial activity against *L. monocytogenes*. A very recent and interesting work by Abbasi et al. [28] reported the dampening effect of Alm ion channel on conductivity by an order of magnitude upon introducing an amiloride molecule, which does not inhibit the ion channel formation itself. Taylor et al. [29] showed that Alm peptides can induce lipid flip-flop even in a surface-bound state by disordering lipids in the membrane.

In this study, alamethicin was chosen as the model peptaibol to establish the use of accelerated MD techniques in correctly elucidating their conformational ensemble and dynamics. We carried out consecutive aMD simulations with increasing boost parameters to observe the folding progress in comparison to the experimental structure. The highest boost parameters were chosen to observe Alm folding from an unfolded starting structure achieved within 1 µs. The same procedure can be applied for other peptaibols of unknown three-dimensional structure to obtain structural information within a short amount of time. We also discuss the structural convergence of these simulations. The reweighted free energy landscapes have been produced to understand peptaibol folding dynamics.

Reweighting of all distributions was carried out using the Maclaurin series expansion, which is equivalent to cumulant expansion of first order. Miao et al. [30] state that the Maclaurin series expansion method suppresses energetic noise but may give incorrect energy minimum positions on the free energy landscapes. Another method known as exponential average reweighting, which is equivalent to cumulant expansion of third order, may lead to higher energetic fluctuations. They emphasized the accuracy of cumulant expansion of second order as a reweighting method, especially if the boost potential follows close to a Gaussian distribution. However, Jing et al. [31] argued that the boost potential should follow the Gaussian distribution exactly for cumulant expansion of second order to accurately reweight free energy profiles. Along these developments, Miao et al. [32] proposed the Gaussian accelerated MD (GaMD) technique, in which the boost potential is made to follow a Gaussian distribution and accurate reweighting can be achieved through cumulant expansion of second order. It is an update to the existing accelerated MD technique in a way that results in recovery of distinct low energy states that may be lost in the former technique. During this work, we also carried out three consecutive 300 ns long GaMD simulations on Alm F30/3 for comparison, but slight folding could only be observed for the N-terminus and it took longer time. We agree that the lack of more advanced processing units and deeper understanding of the GaMD technique at our end may have been the reason for unsatisfactory results. Nevertheless, we are in the process of optimizing peptaibol structure elucidation using GaMD and its advanced variants like Replica Exchange GaMD [33]. Furthermore, to overcome the barrier of loss of distinct low energy conformations, we carried out the first aMD simulation with low boost parameters which were increased for the other three simulations. Therefore, the combined trajectory likely traverses the whole dynamic pathway of Alm F30/3 folding. The two major conformations obtained through these simulations and their functional significance with respect to previous studies is also discussed in detail. Furthermore, we also show the use of aMD to model Alm F30/3 hexameric pore embedded in an *E. coli* membrane mimic under the application of an external electric field. Even though the free energy landscapes have not been reported for membrane-peptide simulations, no significant deformations were observed due to the application of boost potential. The use of aMD has been highlighted to study such systems within a short period of time with efficacy.

## 2. Results and Discussion

### 2.1. Clustering of Three Consecutive aMD Simulations and Comparison with Fourth Simulation: Free Energy Landscapes

**Figure 1:**
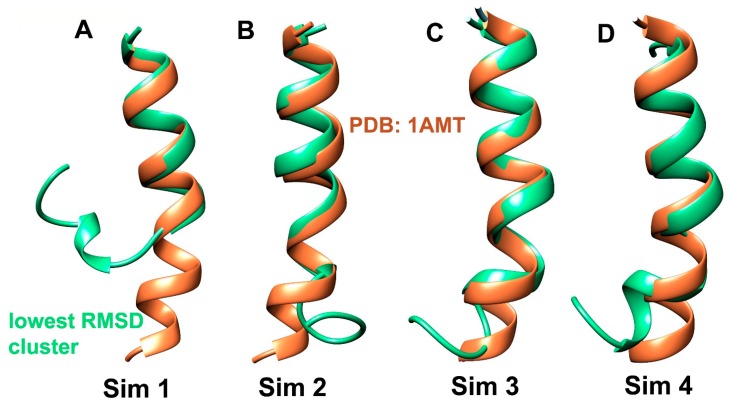
The representative structures of different clusters obtained for individual simulation chosen based on lowest RMSD with X-ray crystallographic Alm F30/3 structure available from PDB: 1AMT.

The unfolded conformation of Alm F30/3 was used as a starting point for three consecutive ~900 ns (refer to Table 1 for exact timescales) long simulations with increasing “boost” parameters. The first simulation (Sim 1) was carried out with a1, a2 = 0.16 and b1, b2 = 4 which revealed successful folding of the N-terminal segment (Aib1-Leu12) but an incomplete folding of the C-terminus as shown through superimposition of the representative structure of the energy minimum and experimentally known structure (PDB ID: 1AMT) with backbone root-mean-square deviation (RMSD) value of 5.02 Å in Figure 1A. The next simulation (Sim 2) was started from this point with slightly higher boost parameters of a1, a2 = 0.20 and b1, b2 = 4.5 to observe the time length of achieving complete peptide folding. This 950 ns long aMD simulation was clustered into three groups, out of which cluster 2 was closest to the experimental structure (Figure 1B) with RMSD of 1.87 Å. At this point, it was deemed a better choice not to increase the boost further as it may interfere with correct reweighting of energy distribution. These boost parameters were deemed appropriate for fast folding. A third ~900 ns long simulation (Sim 3) was carried out at this point which achieved complete C-terminal folding. The clustering resulted in the most populated cluster whose representative structure is the closest to PDB conformation with a RMSD value of 1.51 Å (Figure 1C). The three trajectories were later combined for most of the analysis. The reweighted torsional (phi-psi) angle distribution for each residue calculated for all these 3 simulations have been provided and discussed in Supplementary Figure S1.

After carrying out three consecutive aMD simulations with increasing boost parameters, we were also curious to observe the extent of folding observed using these boost parameters with a completely unfolded Alm configuration as the starting structure. A separate 1 μs long simulation (Sim 4) was carried out that resulted in highly folded structures with both bent and linear configurations. This simulation shows that slightly aggressive boost parameters used for ~1 μs long aMD carried out using GPUs are sufficient for folding simulations of such short peptaibols. Bucci et al. [34] also reasoned that 1 μs long aMD simulations are sufficient for folding simulations of their modified tripeptide. The representative structure of cluster 5 is closer to the experimental structure with an RMSD value of 1.8 Å between them (Figure 1D). 

In conclusion, the combined trajectory of the first three simulations is comparable to the fourth ~1 μs long simulation carried out with aggressive boost parameters. Complete Alm F30/3 folding was achieved within 1 μs, which indicates that this procedure is apt to elucidate short peptaibol structure in a short period of time.

**Figure 2:**
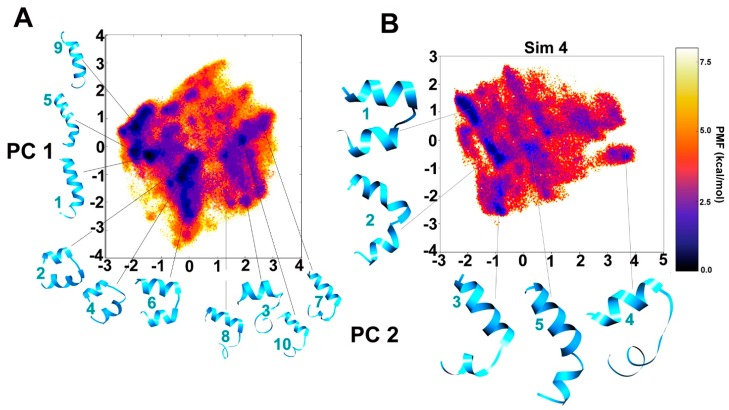
Reweighted potential of mean force (PMF) free energy landscapes of the first two principal components (PCs) calculated from dihedral angles, phi-psi, for better clustering based on internal motions. The deepest blue regions indicate energy minimum. The representative structures of each cluster are provided for (**A**) combined 3 simulations, (**B**) the 4th simulation. The cluster numbers are given in cyan.

The dihedral principal component analysis (PCA) based free energy landscape (FEL) between PC1 and PC2 has been provided for the combined trajectory of the first three simulations clustered in 10 major representative groups (Figure 2A). The darkest violet regions on the FEL map (Figure 2) show the lowest energy conformation clusters which denote the metastable states of Alm F30/3. As it can be observed on the reweighted maps, at least three distinct clusters can be identified in local energy minimum regions, i.e., the linear helical form (clusters 1, 5 and 9), the bent form (clusters 2, 4 and 6) and the incompletely folded (clusters 3, 7, 8 and 10) conformations. The inter-conversion between the bent and linear forms seems to be energetically allowed within 2 kcal mol^–1^ and the jump between these two is achieved multiple times throughout the trajectory. The C-terminus requires longer time than the N-terminus to completely sample the folded helical configuration. Similarly, the fourth trajectory has been separately projected on to dihedral-based PCs in Figure 2B to avoid missing low energy conformations obtained during previous simulations. The bent conformations of cluster 1 and 2 appear interchangeably for about ~10% and ~8% of 1 μs long simulation trajectory, respectively, roughly from 300 to 800 ns and form the most populated conformational group. The conformation of cluster 5 is closest to the experimental structure. The dihedral angle based FEL plots of the combined trajectory from the first three simulations in comparison with the fourth 1 µs long trajectory shows that a similar conformational space could be covered in less time if slightly aggressive boost parameters are used. However, it comes with the risk of missing distinct low energy conformations. For this reason, it is advisable to carry out few simulations with smaller boost potential.

**Figure 3:**
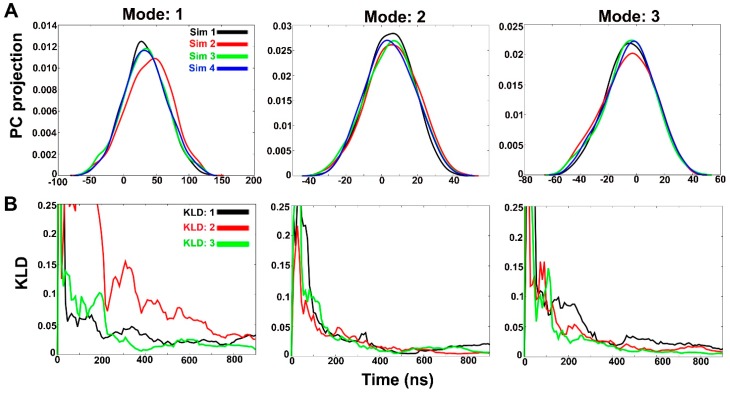
(**A**) Histograms of projection of principal components 1, 2 and 3 for all four simulations in water. Histograms were calculated using a Gaussian kernel density estimator. (**B**) A measure of overlap between histograms from independent simulations calculated using Kullback-Leibler divergence method. The slope values lying below 0.025 indicate convergence between two independent runs.

In order to observe structural convergence amongst the three independent simulations, the Kullback-Leibler divergence (KLD) was calculated to measure the extent of overlap between probability distribution [35]. This probability distribution is based on PCA of the system Cartesian coordinates instead of dihedral angles. The trajectories are named as “Sim 1” to “Sim 4” and the extent of PC overlap signifies convergence between independent runs. The degree of PC overlap suggests that the independent simulations sampled similar conformational space, hence, convergence. It is based on the idea that any two simulations will eventually sample the same phase space even when started from different starting structures as a measure of true convergence. Figure 3A shows the histograms of projection of coordinates along the first three eigenvectors or modes (accounting for 53% overall motion). The first three PCs obtained from the combined trajectory account for 29%, 16% and 8% (in same order) of overall motions. In case of all PC histograms, the best overlap can be observed between Sim 1, Sim 3 and Sim 4 in comparison to Sim 2 projection which signifies that Sim 2 undergoes a slightly different folding pathway. A better overlap can be observed for all trajectories in case of modes 2 and 3. This divergence can be quantified by calculating KLD vs time (ns).

Figure 3B shows KLD between subsequent histograms as a function of time from four simulations for PCs 1, 2 and 3. KLD:1 is divergence between Sim 1 and Sim 2, KLD:2 between Sim 2 and Sim 3 and KLD:3 between Sim 3 and Sim 4. The rapidly decreasing slope of KLD vs time of any two trajectories indicates the reduction in divergence between sampled conformations. We chose the KLD value of 0.025 as cutoff for convergence based on a previous study [36]. As evident from Figure 3B, convergence for Mode 1 for all four trajectories, except Sim 2, is obtained only after 700 ns. KLD:2 (divergence between first PC of Sim 2 and Sim 3) shows the highest divergence for Mode 1 even though both simulations were started with the same boost parameters but different starting structures.

This also indicates that Sim 2 undergoes a different path of Alm F30/3 folding. KLD:3 (divergence between first PC of Sim 3 and Sim 4) reaches the threshold at ~400 ns. The two trajectories, the former with a semi-folded starting structure and the latter with completely unfolded structure, evolve quickly and sample similar configurations to achieve close to experimental Alm F30/3 structure. In case of Mode 2 and Mode 3, the convergence between all three trajectories is reached at ~400 ns time scale. Using boost parameters a1, a2 = 0.20 and b1, b2 = 4.5 for ~1 μs proved to be appropriate to achieve the near-native Alm F30/3 conformation starting with the unfolded Alm F30/3 structure. A similar approach can be adopted to model other peptaibols of unknown structure. The convergence of all simulations was proven based on the Kullback-Leibler method which showed that Sim 2 follows the most divergent path of conformational folding and takes longer timescales to converge, while the other three show structural convergence within 400 ns.

### 2.2. Alamethicin Backbone Bending: Functional Importance?

In the previous section we observed at least two very distinct Alm F30/3 conformations: linear and bent helices. Here we discuss whether there is a functional importance of such dynamic structural shift. This knowledge may direct us to understand how these peptides show membrane-perturbing properties. Using paramagnetic enhancements of nuclear relaxation, North et al. [37] demonstrated that the Alm backbone undergoes large structural fluctuations that result in shorter distances between the C-terminus and various positions along the backbone. They linked this observation with the voltage-gating mechanism of the Alm channel. A previous study by Franklin et al. [38] showed that simulated annealing with NMR restraints of Alm peptide bound to micelles yielded both bent and linear conformations, which prompted them to confirm their analysis by appending a spin label to one of the bent conformations and energy minimization using the steepest descent method. The same bent conformation was obtained as the energy minimum each time. Comparing these observations with previous studies, North et al. [39] reasoned that Alm must be in a dynamic equilibrium of linear and bent conformations and that it may provide the “conformational switch” of voltage gating in the Alm channel. In other words, the bent/closed form of Alm bound to membrane may indicate the absence of transmembrane voltage, which—when applied—would allow conversion to the linear and amphipathic Alm conformation. Gibbs et al. [40] reported on the phenomenon of helical bending around residues Aib10-Aib13 of Alm observed during 1 ns long simulation in methanol. The structural states obtained had either Aib10 or Gly11 carbonyl group oriented away from the backbone and did not seem to greatly affect adjacent helix structure. The functional role of helical bending in channel formation was hypothesized. 

**Figure 4:**
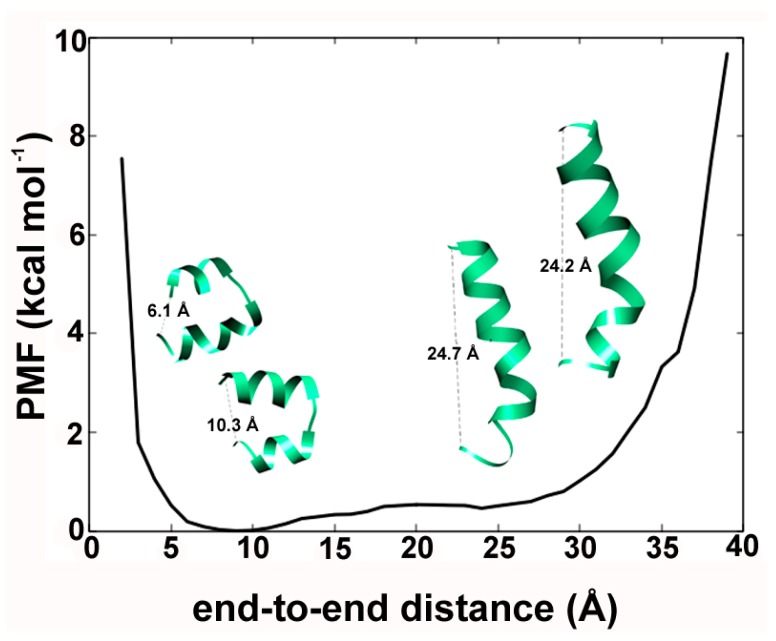
The reweighted potential-of-mean-force values in kcal mol^−1^ as a function of end-to-end distance of Alm calculated for each step in the combined trajectory. The energy minimum lies for a distance value of 10 Å which denotes a highly bent backbone.

We were curious to observe the phenomenon of helical bending obtained through aMD. The end-to-end distance (in Å) between the first residue Aib1 (N-terminus) and Pol20 (C-terminus) was calculated for each frame of the combined trajectory. This data was used to calculate the PMF (in kcal mol^–1^), which is simply the change in free energy as a function of any reaction coordinate. The PMF describes the energy minimum as the most stable state along that function. The end-to-end distance values of 9 and 10 Å designated by highly bent helical conformations (Figure 4) indicate their stability. The distance values of ~25 Å indicated by linear Alm backbone conformations are easily accessible with under ~1 kcal mol^–1^ of energy boost. It supports the idea of a dynamic equilibrium between the two conformations in line with previous studies [39]. Similar results were also obtained in our previous studies with other fungal peptaibols for example, trikoningin KA V [41], the energy minimum conformation of tripleurin XIIc obtained in the hydrophobic solvent chloroform [36], paracelsins B and H, and the newly discovered brevicelsin I and IV molecules [42]. This movement along the backbone from bent to linear conformation is the largest scale of motion as described by the first mode calculated through normal mode wizard (Supplementary Video 1). This indicates that peptaibols like Alm are capable of adjusting their backbone bend in response to bilayer thickness. 

The hairpin-like helical conformation, which is representative for clusters 2, 4 and 6 (Figure 2) can be explained due to the presence of the glycine residue at the R11 position. The N-terminal helical continuity in Alm F30/3 always breaks at the Gly11 position. Högel et al. [43] systematically described the local helix bending observed at the glycine position that effectively perturbs the conformational flexibility in transmembrane helices. Glycine does not have a side-chain and can easily conform into many energetically stable Φ-Ψ torsional states as can be seen on the reweighted energy landscape of Gly11 in Supplementary Figure S1. We calculated the PMF for end-to-end distance to find out the lowest energy conformation amongst the bent and linear. The results clearly show that the bent helix lies in the true energy minimum while the linear is easily accessible. It was correlated with the strategic placement of bend-inducing amino acid residues like proline and glycine found almost at the same position in almost all peptaibol sequences.

### 2.3. Alamethicin Hexamer Pore in a Bacterial Mimicking Bilayer Membrane

**Figure 5:**
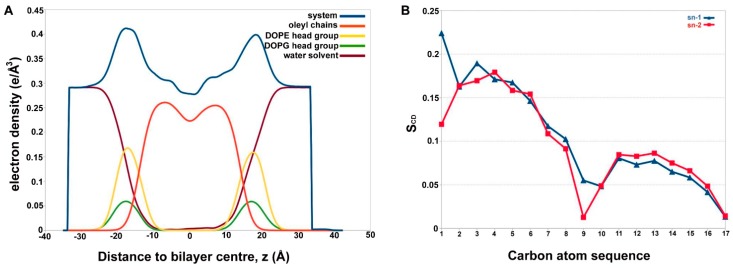
(**A**) The electron density profile for each constituent of membrane system and water calculated across the bilayer normal (Z-direction). A slight rise in the profile of water around 10 Å indicates presence of water in Alm F30/3 pore. Total electron density (in blue), the water density (brick red), the acyl tails of DOPE lipids (orange), the phosphoethanolamine lipid heads of DOPE (yellow) and phosphoglycerol heads of DOPG (green), (**B**) lipid order parameters of the acyl chains, |S_CD_| can be compared with previous experimental values. The acyl chains show high disorder that may be a result of presence of the Alm F30/3 pore.

It is a well-known fact that Alm shows multiple conductance states, which directly correlates with the number of peptide monomer units. A previous study performed to highlight the difference in channel conductance with quadromer, pentamer, hexamer, octamer and even nonamer Alm F30/3 pores highlighted the occluded state of 3-mer and 4-mer pores, while pores beyond 5-mer were comparable [16]. The hexamer Alm pore is the most widely accepted model of its channel formation. For this reason, we chose to simulate a hexamer Alm model in 1,2-dioleoyl-*sn*-glycero-3-phosphoethanolamine: 1,2-dioleoyl-*sn*-glycero-3-[phospho-rac-(1-glycerol)] (DOPE:DOPG) 3:1 bilayer membrane, a simplistic representation of *E. coli* membrane [44]. DOPE is a cationic or neutral lipid, whereas, DOPG is negatively charged. The electron density profiles for DOPE: DOPG lipids and water calculated across the bilayer normal (Z-direction) has been provided in Figure 5A. A slight increase in water density from 0 to 10 Å in the bilayer signifies water displacement through the Alm pore. The lipid order parameters of the acyl chains were also determined, which can be directly compared with experimental S_CD_ values. S_CD_ is a measure of the relative orientation of the C-D bonds with respect to bilayer normal and can be calculated as |S_CD_| = 0.5 <3cos^2^*θ* – 1>, where *θ* is the angle between bilayer normal and the vector joining *C_i_* to its deuterium atom, where <> means average of all lipid molecules. All contributions from conformational disorder, local tilting known as lipid wobble and collective motions constitute the S_CD_ parameter and thus, can be a measure of membrane fluidity [45,46].

Figure 5B shows the average lipid acyl chain order parameters for mixed DOPE and DOPG. It could be compared to the plateau values of the two chains sn1 and sn2 taken from carbon number 4 to 6 for DOPE as 0.211 and 0.215, respectively [47]. The plateau values for sn1 and sn2 in this case (for a DOPE:DOPG mixture) membrane is slightly lower, averaging at ~0.16 for both chains. This could be a result of DOPG mixing or the presence of membrane-perturbing peptide channel. It is evident that the membrane is more disordered than the pure DOPE membrane system. Moreover, another study conducted on *Pseudomonas aeruginosa* mimic membrane system, comprising of DOPE and DOPG with a synthetic lipid, observed an average S_CD_ value for pure DOPE inner membrane as 0.180 and for DOPG as 0.112 [48]. On the other hand, the average value for the membrane system used in this study is 0.10. This clearly shows that the membrane is highly disordered during the course of simulation due to the presence of the Alm F30/3 channel. The primary results of membrane-peptide simulations indicated that the Alm F30/3 hexamer channel increases membrane disorder, which eventually leads to leaking of water molecules and may lead to the disintegration of the bacterial cell. 

The diffusion coefficient (DC) of water inside the Alm F30/3 pore was calculated using mean square displacement (MSD Å^2^ ps^–1^). It is the average distance that all water molecules travel from their starting position in XYZ direction. The MSD was reported along the z-direction. The speed of water movement can be estimated based on the rise in slope of MSD vs. time plot. The diffusion constant is calculated by fitting a slope to the MSD vs. time plot and multiplying it by 10.0/2 × N (where N is the number of dimensions). The diffusion constant was calculated to be 0.0311 cm^2^ s^–1^ and 0.0245 cm^2^ s^–1^ for the first and second simulation, respectively. This in comparison to the value of diffusion of water in water (as a liquid) at 0.000023 cm^2^ s^–1^, is much higher. 

**Figure 6:**
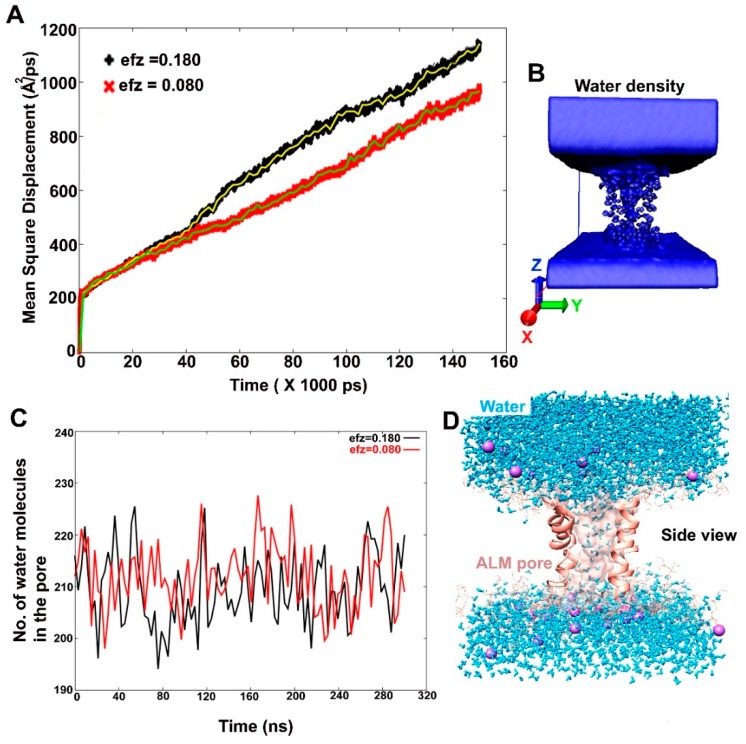
(**A**) The mean square displacement (Å^2^ ps^−1^) values of water calculated as a function of simulation time. Using the slope of MSD curve, the average diffusion coefficient was calculated to be 0.0311 cm^2^ s^−1^ and 0.0245 cm^2^ s^−1^ for Mem-sim1 and Mem-sim2, respectively. Mem-sim1 clearly shows higher displacement of water under the influence of a comparatively stronger external electric field (0.07 V nm^−1^), (**B**) A diagrammatic representation of water density around the pore, (**C**) The number of water molecules present in the Alm F30/3 pore at a given time, (**D**) A cartoon representation of water molecules passing through the Alm F30/3pore. The front two monomers have been hidden for visual clarity.

Conversely, the DC value of water (as gas) in air at 273 K temperature is 0.219 cm^2^ s^–1^. It shows that in membrane simulations, the density of water molecules is more similar to the gaseous phase. Another important fact to note while calculating DC values from simulations is that most force fields, like the TIP3P water model used in this study, overestimate the diffusion coefficient even in bulk solution [49]. It is apparent from DC values for the two trajectories that a higher external electric field value (0.07 V nm^–1^) causes slightly higher MSD of water molecules across z direction (Figure 6A). The density of water molecules in the z-direction is also shown in Figure 6B by histogramming all water O atoms on a grid with spacing 1 Å. The resultant file can be visualized in Visual Molecular Dynamics (VMD) software. The MSD analysis clearly indicated bulk movement of water molecules through the Alm F30/3 hexamer channel.

Figure 6C is a graphical representation of number of water molecules present in a hypothetical shell. This shell was created around the peptide pore residues at a distance of 3.4 Å. Therefore, the number of water molecules present inside the pore are counted as a function of simulation time. It is clear that from 200 to 230 water molecules are always present at any given time. A 14 μs long all-atom classical MD simulation of the Alm hexamer pore in DOPC membrane studied by Perrin Jr. et al. [19] reported about 40 to 50 water molecules at any given time. The difference in this number can be attributed to multiple factors like the application of an external electric field in our case and the use of accelerated dynamics. They calculated the number of water molecules at a distance of 10 Å from the bilayer center forming a 20 Å region in total (to be in the pore), while our calculation includes the complete length of the Alm F30/3 pore (30 Å) and thus, the surface-bound regions and water molecules are also considered. A diagrammatic representation of the presence of water molecules in the channel is shown in Figure 6D. The peptides present in the front view have been hidden for clear visualization. The displacement of water through the channel can also be visualized in the animation provided as Supplementary Video 2. The high number of water molecules present in the pore as a function of simulation time correlates with the high MSD value discussed above.

**Figure 7:**
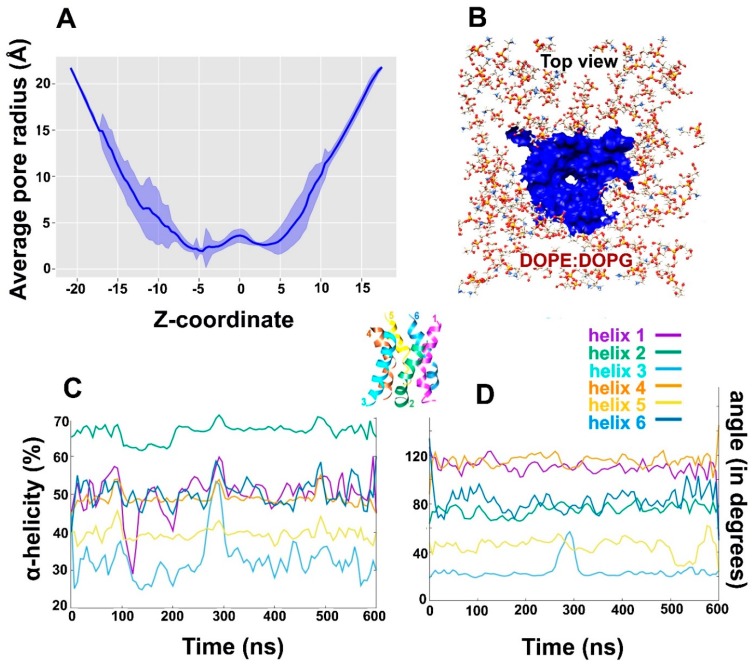
(**A**) The average radius of the Alm F30/3 pore (Å) calculated along transmembrane coordinate. The major fluctuation in the radius is shown by the C-terminus probably to accommodate incoming water flux, (**B**) Cartoon representation of the top view of the Alm F30/3 pore surface in blue, (**C**) percent α-helicity calculated for each Alm F30/3 monomer from the pore with respect to simulation time (in ns), (**D**) the bend angle (calculated as dot product of two vectors passing through the N- and C-terminals of each monomer) as a function of simulation time. Figure 7C,D can be correlated assuming that the higher the bend angle, the lower will be its α-helicity. The helices are color-coded for visual interpretation.

The size of hexamer pore must dynamically change to accommodate the influx of bulk water and thus, pore radius was calculated as function of simulation time. The average pore radius (in Å) as a function of z coordinate was calculated over each frame of the combined 600 ns trajectory (Figure 7A) using HOLE utility from MDAnalysis. It is clear, that the C-terminus (top of Alm F30/3 pore) undergoes stronger deviation than the N-terminus. The funnel-shaped top of the pore undergoes thickening and thinning of pore size continuously. The pore is thinnest at the center of the bilayer. Figure 7B shows the top view of the pore surface surrounded by lipid heads. We were also curious to observe the changes in secondary structure of Alm F30/3 peptides in the hexamer as a function of simulation time. Based on percent α-helicity calculated for each peptide monomer as shown in Figure 7C, it is apparent that helices 1 and 3 undergo major changes. Helices 1, 4 and 6 show an average 50% helicity while helix 2 shows the highest at 65% and helix 3 shows the lowest at 30%. It must be noted that all 6 peptides started with the same conformation and yet undergo vastly varied conformational changes. Perrin Jr. et al. [19] also noted that the average % α-helicity drops to ~47%, which is in line with circular dichroism experiments of Alm channel within DOPC membranes. To correlate this change in percent α-helicity for each monomer, we calculated the angle (in degrees) between a vector passing through the center of mass of N-terminal residues and a vector passing through C-terminal residues (Figure 7D). This angle is mentioned as bend angle from here on. As expected, helix 3 that was observed to have the least percent α-helicity also has the least value of bend angle, followed by helix 5. It means that these monomers undergo extensive backbone bending during simulation, which is not feasible with a strict α-helix conformation. Therefore, these monomers unwind to achieve a more relaxed spiral conformation to accommodate pore transformation. Conversely, helices 1 and 4 show an average α-helicity around 50% and yet their bend angle values are amongst the highest. It is evident that helices 1 and 4 do not undergo drastic backbone bending but lose their helicity indicating towards other factors affecting pore dynamics. One reason could be to accommodate the passage of bulk water molecules. Finally, the last observation regarding helix 2 showing highest α-helicity (65%) but a smaller bend angle of 80° indicates that a certain degree of backbone bend is possible without completely losing α-helicity. The percent helicity for each peptide in the Alm F30/3 was calculated and correlated with their backbone bend angle as a function of time. A highly bent conformation is sterically difficult to achieve with the presence of strict α-helix for the whole sequence. Therefore, few monomers show interconversion between α-helix and turns. On the other hand, few other monomers show that a certain degree of backbone bend is possible with strict α-helical conformation. 

Alm F30/3 is the most studied molecule in the class of peptaibols, which belong to the antimicrobial peptide (AMP) class. All known antimicrobial peptides are parts of the innate immune responses of all life forms known to us. These may be cationic or anionic in some cases and constitute ~50% of hydrophobic amino acid residues. They can exist in four major structural types, α-helix, β-sheet, hairpin or loop and extended, and may act as membrane-perturbing, pore-forming or immunomodulatory agents. The negatively charged lipid headgroups of bilayer membranes form attractive contacts with cationic AMPs. However, peptaibols lack the presence of positively charged residues and show a surface-bound state before penetrating the membrane in large numbers and form stabilized pores. These pores have peptide backbone carbonyls facing inside that makes it neutral in charge very similar to aquaporin proteins, class of integral membrane proteins, that are also similar in shape [50]. Cationic AMPs, on the other hand, have positively charged side chains lining the pore interior which may make it rigid and narrower and affect water-ion influx. 

Another important factor is the presence of both hydrophobic and hydrophilic residues in the chain, folding in such a way that a hydrophobic and a hydrophilic face are formed. It is implicated during pore formation, where the hydrophobic side faces the transmembrane region and the hydrophilic side faces the pore cavity. In such a way, water molecules can easily leak through the stabilized pore. The amphipathic nature is therefore a crucial structural requirement for such peptides to show transmembrane activity. The structure of aquaporins is formed as a bundle of six transmembrane pores in an hourglass shape which is similar to the hexamer Alm F30/3 pore model [50]. However, aquaporins allow only water molecules and sometimes small solutes to pass through the membrane while peptaibol pores are known to cause leakage by also allowing passage of ions across the membrane along with water. This difference has been attributed to the presence of four aromatic/arginine residues that act as selectivity filters in case of aquaporins. Peptaibols generally lack the presence of positively charged amino acid residues like arginine, histidine and lysine and therefore, do not show selectivity. Therefore, only those polypeptide sequences which show such qualities may lead to similar water influx and membrane disintegration. Peptaibols are a special class of AMPs because of the presence of non-standard amino acid residues, which are produced as secondary metabolites and the peptide sequence is arranged through a large assembly of protein subunits known as non-ribosomal peptide synthetases (NRPSs). Although many general AMPs have been clinically approved to be used as therapeutic agents, for example enfuvirtide [51], oritavancin [52] etc., peptaibols present a promising scope as the next line of defense. This study has been carried out as one of the first steps in understanding the potential of peptaibols as therapeutic agents. The knowledge of peptaibol structure and re-construction of their mode of action shall give crucial insight to exploit their antimicrobial properties.

## 3. Materials and Methods 

### 3.1. Partial Charge Calculation and Force Field Library Generation for Non-standard Residues

As famously known, fungal peptaibols are characterized by their unusual amino acid content. In the Alm F30/3 sequence, Aib and Pheol are non-standard residues. The R.E.D server was used for calculation of their partial charges and creating force field libraries. R.E.D stands for RESP ESP charge derive [53,54]. RESP (restrained electrostatic potential) was used to calculate the charges with a HF/6-31G(d) basis set and Gaussian 09 quantum mechanical program interface [55]. The charges for Aib were calculated along with few other standard amino acids. The charges calculated for standard residues were used to confirm with existing “leap” libraries. For each residue, two conformations, i.e., alpha helix (Φ = −63.8, Ψ = −38.3) and beta sheet or C5 (Φ = −157.2, Ψ = 161.9) were used. The terminal residue phenylalaninol was also parameterized using two molecules, ethyl alcohol and phenylalanine. The Amber “leap” library is provided in Tyagi et al. [36] for both residues. The sequence of Alm F30/3 is: AcAib^1^-Pro^2^-Aib^3^-Ala^4^-Aib^5^-Ala^6^-Gln^7^-Aib^8^-Val^9^-Aib^10^-Gly^11^-Leu^12^-Aib^13^-Pro^14^-Val^15^-Aib^16^-Aib^17^-Glu^18^-Gln^19^-Pheol^20^.

### 3.2. Accelerated Molecular Dynamics Simulations of Alamethicin

The unfolded Alm F30/3 peptide conformation was prepared by using the ‘tleap’ module of AmberTools18 and solvated in water (TIP3P water model) as solvent. In total, 4701 water molecules were added with a box size of 48.79 × 71.80 × 54.41 Å and a volume of 190680.596 Å^3^. Amberff14SB force field was used to prepare and minimize the system [56]. It was prepared for aMD in six consecutive steps as described by Tyagi et al. 2019 [36]. The unfolded alamethicin peptide was simulated in water using aMD for ~900 ns consecutively with three independent starting structures (~900 ns × 3) making a combined simulation time of 2.7 µs. All simulations were carried out at 300 K temperature, 2 fs time step, and energies and boost information were recorded at every 1000 steps. The electrostatic interactions were calculated using PME (particle mesh Ewald summation) [57] long-range interactions were also calculated with cutoff of 10.0. The temperature scaling was carried out using Langevin thermostat without pressure scaling. SHAKE algorithm was applied on all bonds involving hydrogen. The GPU machines available through the NIIF clusters of Hungary were utilized for all aMD simulations using *pmemd.cuda* implementation of Amber14. aMD can be carried out using three criteria, (i) the whole potential at once (iamd = 1) or (ii) independently boosting the torsional terms of the potential (iamd = 2), and (iii) to boost the whole potential with an extra boost to torsions (iamd = 3). The third criterion seemed to be an appropriate choice, as the dual boost option provides a better reweighting distribution. The extra parameters E_dihed_, α_dihed_, E_total_ and α_total_ were calculated as required in Equation (1):

(1) Edihed=Vavgdihed+a1×Nres, αdihed=a2×Nres5Etotal=Vavg_total+b1×Natoms, αtotal=b2×Natoms

where *N_res_* is the number of peptide residues (21, with an addition of acetyl group at the N-terminal), *N_atoms_* is the total number of atoms in the system which is 14391. *V_avg_dihed_* and *V_avg_total_* are average dihedral and total potential energies obtained from the 100 ns long cMD run. The various parameters used for all aMD simulations have been summarized in Table 1 The reweighting procedure and analysis details have been adopted from Tyagi et al. [36].

### 3.3. Accelerated Molecular Dynamics Simulations of Alamethicin Pore in DOPC Bilayer Membrane

The hexamer pore of Alm F30/3 peptide was obtained through M-ZDOCK server (http://zdock.umassmed.edu/m-zdock/) [58]. This is a Fast Fourier Transform based protein docking program that predicts the structures of cyclically symmetric multimers. The hexameric pore was then embedded into a 3:1 mixture of DOPE and DOPG bilayer membranes which mimics a bacterial (*E. coli*) membrane constitution. This system can be easily prepared in an Amber-ready format by using the ‘*packmol-memgen*’ [59] workflow available with AmberTools18 that uses ‘*Memembed*’ [60] to obtain pre-oriented protein conformation with respect to the membrane. This system was solvated in 4410 water residues. The Alm F30/3 hexamer channel embedded in DOPE:DOPG bilayer system was prepared for aMD simulations starting with minimization followed by two steps of system heating and 10 steps of equilibration. This system was energy minimized for 10,000 steps which switches to conjugate gradient method after 5000 steps of the steepest descent method. The minimization was done at constant volume, no SHAKE algorithm was applied, and the non-bonded cutoff was set to 10.0 Å. The minimized system was set for two rounds of gradual ‘heating’ to reach the ‘production run’ temperature. In the first step, the system was heated to 100 K while the second step reaches a temperature of 303 K. The 10-step equilibration was carried out at 303 K temperature for 500 ps each. A short 25 ns long production run at 303 K was carried out with constraints on bond distances to calculate the aMD boost parameters followed by two consecutive aMD simulations of 300 ns each with 2 ps time step. Each aMD simulation was carried out with dual boost (iamd=3) option at 300 K temperature regulated using a Langevin thermostat (Table 1). A weak external static electric field was also applied along the z direction (across membrane) with a value of efz (intensity in kcal (mol × A × e)^−1^) = 0.180 and 0.080 for the first and second simulations, respectively [61]. The values of efz were chosen in a way that the magnitude of resulting electric potential is slightly higher than the voltage across plasma membrane. A membrane with 35 Å thickness has a potential of ~70 millivolts (mV) which is 0.07 V per 3.5 × 10^−7^ cm or 0.02 V nm^−1^ [62]. A 0.180 kcal (mol × A × e)^−1^ translates to an electric potential of 0.07 V/nm while 0.080 kcal (mol × A × e)^−1^ translates to 0.03 V × nm^−1^. A distance restraint was applied for all glutamine amino acid residues owing to their importance in Alm F30/3 pore stability. The average pore radius was calculated using the HOLE utility [63] and water density available through MDAnalysis [64,65].

## 4. Conclusions

This study highlights the use of enhanced sampling molecular dynamics technique known as accelerated MD (aMD) to elucidate fungal peptaibol structures. The gap between the knowledge of these sequences and their structure is ever increasing which requires efficient and cost-effective computational methods to be fulfilled. Through a series of aMD simulations we show that ~1 µs time scale with slightly aggressive boost parameters is appropriate to completely uncover peptide dynamics. The experimental structure of alamethicin F30/3 is compared with representative structures of highly populated clusters in each simulation. aMD was also used to recreate the Alm F30/3 hexamer-membrane system by using *E. coli* membrane mimic under a weak electric field. The various quantities were compared with previous experiments, which suggested that the presence of the Alm F30/3 pore results in membrane disorder and leads to a large influx of water molecules through the channel. The C-terminus seems to fluctuate resulting in changes of the pore size throughout the simulation. The water influx is increased under the application of an external electric field. Overall, this study shows that aMD can be successfully used to elucidate folding dynamics of small, bioactive peptides and to simulate their mode of action over bacteria mimicking membrane systems.

## Figures and Tables

**Table 1:** Summary of various accelerated molecular dynamics parameters.

Simulations	Starting Conformation	Time (ns)	a1,a2 Total (kcal mol^−1^)	b1,b2 Dihedral (kcal mol^−1^)	Avg Boost (kcal mol^−1^)
**Sim 1**	Unfolded	936	0.16	4	11.21
**Sim 2**	Folded N-terminal	950	0.20	4.5	11.10
**Sim 3**	Folded with bent backbone	897	0.20	4.5	29.85
**Sim 4**	unfolded	1000	0.20	4.5	24.09
**Membrane-sim 1**	Hexamer with efz = 0.180	300	0.16	4	31.71
**Membrane-sim 2**	Hexamer with efz = 0.080	300	0.16	4	22.60

## Supplementary Materials

The following are available online at https://www.mdpi.com/1422-0067/20/17/4268/s1.
