# Correction: Tyagi, C., et al. Accelerated Molecular Dynamics Applied to the Peptaibol Folding Problem. *International Journal of Molecular Sciences*, 2019, *20*, 4268

**DOI:** 10.3390/ijms22073707

**Published:** 2021-04-02

**Authors:** Chetna Tyagi, Tamás Marik, Csaba Vágvölgyi, László Kredics, Ferenc Ötvös

**Affiliations:** 1 Department of Microbiology, Faculty of Science and Informatics, University of Szeged, Szeged, Közép fasor 52, H-6726 Szeged, Hungary; mariktamas88@gmail.com (T.M.); mucor1959@gmail.com (C.V.); kredics@bio.u-szeged.hu (L.K.); 2 Doctoral School of Biology, Faculty of Science and Informatics, University of Szeged, Szeged, Közép fasor 52, H-6726 Szeged, Hungary; 3 Institute of Biochemistry, Biological Research Centre, Szeged, Temesvári krt. 62., H-6726 Szeged, Hungary; otvos@brc.hu

The authors wish to make the following corrections to this paper [1]:

The authors have realized an unfortunate error in the reporting of diffusion coefficient values in the legend of Figure 6A. The corresponding text says “The diffusion constant was calculated to be 0.0311 cm^2^ s^−1^ and 0.0245 cm^2^ s^−1^ for the first and second simulation, respectively. This is in comparison to the value of diffusion of water in water (as a liquid) at 0.000023 cm^2^ s^−1^, is much higher.”, which should be changed to “The diffusion constant was calculated to be 0.311 × 10^−5^ cm^2^ s^−1^ and 0.245 × 10^−5^ cm^2^ s^−1^ for the first and second simulation, respectively. This is much lower in comparison to the value of 0.23 × 10^−4^ cm^2^ s^−1^ for diffusion of water as a liquid, which is expected as the behaviour of water molecules in ion channel changes drastically in comparison to bulk water.”

Consequently, the following sentences “Conversely, the DC value of water (as gas) in air at 273 K temperature is 0.219 cm^2^ s^−1^. It shows that in membrane simulations, the density of water molecules is more similar to the gaseous phase.” are also irrelevant and incorrect, which should be removed.

The authors declare that this change reflects a small part of the overall results and does not affect the validity of other results discussed in the paper. The authors would like to apologize for any inconvenience caused to the readers by these changes.
