# Supplementary material for: Accelerated Molecular Dynamics Applied to the Peptaibol Folding Problem
Source: Int J Mol Sci. 2019 Aug 30;20(17):4268. doi: 10.3390/ijms20174268 (PMC6747184; doi:10.3390/ijms20174268)
Supplement: Supplementary file 1 [file ijms-20-04268-s001.zip › ijms-574596 supplementary.pdf]

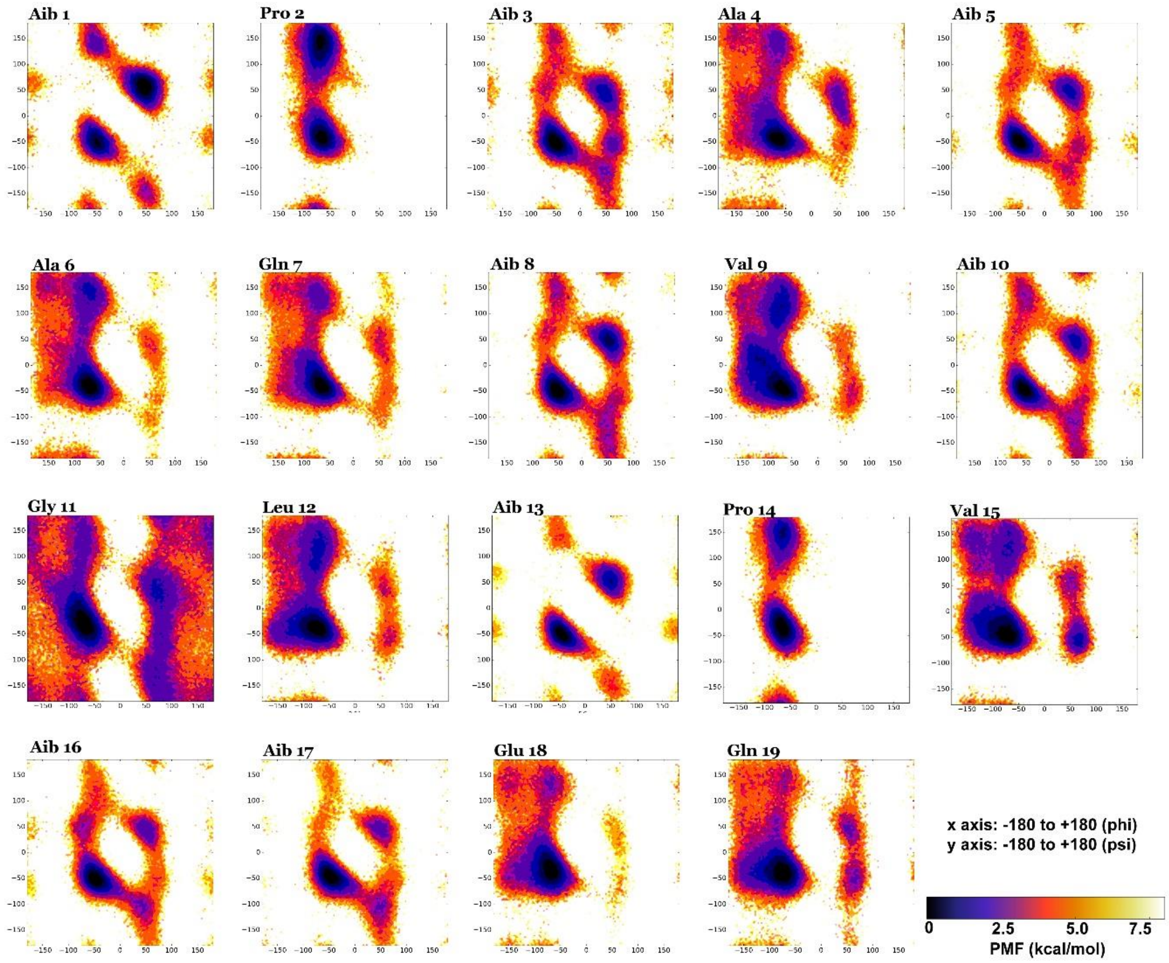

Figure S1. The reweighted phi-psi torsional distribution for each residue averaged over the three 900 ns simulations. As can be observed, most residues show an energy minimum in the α-helical region. All Aib residues shift between right-handed and left-handed helical regions owing to their achiral nature. The Gly 11 residue covers a huge area on the plot owing to the absence of a side-chain. The distribution was reweighted using the Maclaurin expansion method.

Video S1. A visual depiction of the conformational change in the backbone of Alm peptide along the first normal mode calculated from aMD simulations by PCA. Normal Mode Analysis is used to track large scale motions in biomolecules that may be functionally relevant. The biggest motion is along the center of the backbone that may render the peptide in either bent or linear configurations. The size and direction of porcupine arrows signify the magnitude and direction of motion. It is clear that the two terminals show the biggest fluctuations, which can be correlated with the average pore radius profile from Figure 7A.

Video S2. An animation created from the two Alm F30/3 hexamer pore and membrane simulations. The lipid molecules have been hidden for clarity. The water molecules easily enter the pore and pass through while few peptide monomers seem to have lost α-helicity at the C-terminus (top of the pore).
